# A CT60G>A polymorphism in the *CTLA-4* gene of the recipient may confer susceptibility to acute graft versus host disease after allogeneic hematopoietic stem cell transplantation

**DOI:** 10.1007/s00251-015-0840-7

**Published:** 2015-05-05

**Authors:** Lidia Karabon, Miroslaw Markiewicz, Anna Partyka, Edyta Pawlak-Adamska, Anna Tomkiewicz, Monika Dzierzak-Mietla, Slawomira Kyrcz-Krzemien, Irena Frydecka

**Affiliations:** Department of Experimental Therapy, L. Hirszfeld Institute of Immunology & Experimental Therapy, Polish Academy of Science, R. Weigl 12, 53-114 Wroclaw, Poland; Department and Clinic of Urology, Wroclaw Medical University, Borowska 213, 50-556 Wroclaw, Poland; Department of Hematology and Bone Marrow Transplantation, School of Medicine in Katowice, Dabrowskiego 25, 40-032 Katowice, Poland

**Keywords:** CTLA-4, Gene polymorphism, Hematopoietic stem cell transplantation, Acute graft versus host disease

## Abstract

T cell activation plays a crucial role in the development of acute graft versus host disease (aGvHD). Cytotoxic T cell antigen-4 (CTLA-4) is a co-inhibitory molecule that negatively regulates T cell activation, differentiation, and proliferation. Single-nucleotide polymorphisms (SNPs) in *CTLA-4* gene may affect its function. Inconsistent observations have been reported regarding the associations of *CTLA-4* SNPs with complications after hematopoietic stem cell transplantation (HSCT). Moreover, the majority of the observations were focused on the donors’ SNPs. Recently, a few studies have shown that recipients’ genetic variations in the *CTLA-4* gene might influence HSCT results. The aim of our study was to determine the influence of the *CTLA-4* gene polymorphisms of the donors and the recipients on the outcome of HSCT. Altogether, 312 donor-recipient pairs were genotyped for the *CTLA-4*c.49A>G (rs231775) and CT60G>A (rs3087243) SNPs using the TaqMan®SNP Genotyping Assays. In this study, it was shown that the recipients’ CT60G>A[GG] genotype, the myeloablative conditioning regimen, and HSCT from an unrelated donor were independent aGvHD risk factors (odds ratio (OR) 2.63, 95 % confidence intervals (95 % CI) 1.45–4.59, *p* = 0.001; OR 2.68, 95 % CI 1.65–4.07, *p* = 0.00003; and OR 1.87, 95 % CI 1.02–3.24, *p* = 0.04, respectively). Moreover, haplotype analysis revealed that possessing allele A in both of the SNPs decreased the risk of aGvHD approximately 1.5-fold (RR 0.69, *p* = 0.008). Our data suggest that the CT60G>A[GG] genotype in the recipient has an impact on aGvHD development, especially in patients receiving transplants from unrelated donors together with the myeloablative conditioning regimen.

## Introduction

Allogeneic hematopoietic stem cell transplantation (HSCT) is used as a curative therapy for a large number of malignant and non-malignant hematologic diseases. One of the major complications after HSCT remains acute graft versus host disease (GvHD), with its occurrence and severity being directly related to the degree of human leukocyte antigen (HLA) incompatibility. The recognition of foreign HLA antigens is the first and key element for T cell activation, but the effective activation requires a second signal, termed co-stimulation (Frauwirth and Thompson 2002). CD28 is the primary T cell co-stimulatory molecule, and it regulates T cell differentiation, cytokine secretion, proliferation, and survival (Salomon and Bluestone 2001). Cytotoxic T cell antigen-4 (CTLA-4), another member of the CD28 family, plays an inhibitory role in the early and late stages of T cell activation (Walunas et al. 1994). Ligation of CTLA-4 with its ligands, CD80 or CD86, inhibits the cell cycle and the activity of the transcription factors NF-κΒ, NF-AT, and AP-1 (Valk et al. 2008).

Because protein synthesis depends on the rate of gene transcription and/or translation, polymorphisms existing in functional sites of genes may affect their expression and function. Several polymorphisms of the *CTLA-4* gene have been found to influence the expression level and function of the CTLA-4 protein and to dysregulate the trafficking of CTLA-4 within cellular compartments (Anjos et al. 2002;Chistiakov et al. 2006;Kouki et al. 2000;Ligers et al. 2001;Wang et al. 2002). Moreover, the *CTLA-4* gene has been described as a susceptibility locus for autoimmune and neoplastic diseases (Chistiakov and Turakulov 2003;Gough et al. 2005;Kavvoura et al. 2007;Ueda et al. 2003). An increasing number of studies have demonstrated an important role for polymorphisms in genes encoding co-stimulatory molecules in organ transplantation (de Reuver et al. 2003;Gorgi et al. 2006;Wisniewski et al. 2006) and HSCT outcomes (Azarian et al. 2007;Perez-Garcia et al. 2007; Sellami et al. 2011;Vannucchi et al. 2007).

Most of the HSCT studies have been focused on the donor *CTLA-4*c.49A>G and CT60G>A gene polymorphisms and the HSCT outcomes, and the results presented in those studies were inconsistent. However, the functional role of those single-nucleotide polymorphisms (SNPs) is well established.

The *CTLA-4*c.49A>G transition causes a Thr/Ala substitution in the leader peptide, which affects the inhibitory function of CTLA-4 by influencing the rates of endocytosis, surface trafficking, and intracellular/surface partitioning (Anjos et al. 2002;Kouki et al. 2000;Ligers et al. 2001).

Several reports indicate that presence of the G allele in the CT60G>A SNP in the 3′UTR of the *CTLA-4* gene is associated with the production of less messenger RNA (mRNA) for the soluble isoform of CTLA-4 (sCTLA-4) compared with the full-length form (fCTLA-4) (Perez-Garcia et al. 2007;Ueda et al. 2003). Therefore, it is hypothesized that sCTLA-4 blocks the B7 binding by the fCTLA-4 isoform presented on the surface of T cells and in this way prevents the transduction of inhibitory signals and leads to the impaired inactivation of T-cells.

Xiao et al. (Xiao et al. 2012) showed that upon stimulation, peripheral blood mononuclear cells (PBMCs) carrying the CT60G>A[GG] genotype exhibited significantly lower proliferation than PBMCs carrying the CT60G>A[AA]/[GA] genotypes.

Our earlier study of protein expression showed that the percentage of cells expressing membrane CTLA-4 and cytoplasmic CTLA-4 in multiple sclerosis patients with the relapsing-remitting form of the disease was higher for individuals possessing CT60G>A[A+] alleles than those with the CT60G>A[GG] genotype (Karabon et al. 2009).

Because donor T cells play the crucial role in acute graft versus host disease (aGvHD) induction, the research on the association between *CTLA-4* gene polymorphisms and HSCT outcomes was focused mainly on the polymorphisms in the HSCT donors. However, recently, a few studies of the influence of the recipient *CTLA-4* polymorphisms on the HSCT outcome appeared in the literature (Mossallam and Samra 2013;Orru et al. 2012;Piccioli et al. 2010;Xiao et al. 2012).

In addition, our recent functional studies indicate that the level of CTLA-4 in the recipient before transplantation affects the aGvHD risk (Karabon et al. 2015).

Therefore, the aim of the present study was to evaluate the association between two polymorphisms: *CTLA-4*c.49A>G and CT60G>A in donors and recipients and the risk of aGvHD.

## Patients and methods

### Patients

Altogether, 312 patients (146 F/166 M, median age 33 years, range 18–57) transplanted in the Department of Hematology and Bone Marrow Transplantation, Medical University of Silesia, Katowice, between 2006 and 2010 and their donors were included in this study.

Of the recipients, 108 underwent related donor (RD) HSCTs, and 204 underwent unrelated donor (URD) HSCTs. For nine recipients, a second transplantation was needed (the second transplantation was RD in one case and URD in eight cases).

Primary diagnoses were acute myeloid leukemia (148), acute lymphoblastic leukemia (66), myelodysplastic syndrome (19), chronic myeloid leukemia (18), severe aplastic anemia (17), paroxysmal nocturnal hemoglobinuria (11), and other cases (33).

The source of the hematopoietic stem cells was bone marrow (BM) in 133 transplants, peripheral blood progenitor cells (PBPC) in 171 cases, and both in 8 cases. For double transplantations, the first source was BM, and the second was PBPC.

Myeloablative conditioning (MAC) regimens were based on cyclophosphamide with either busulfan or total body irradiation (TBI), or on melphalan, fludarabine, and alemtuzumab. Reduced toxicity myeloablative conditioning (RTMAC) was based on treosulfan and fludarabine or cyclophosphamide. Reduced-intensity conditioning (RIC) consisted of fludarabine and busulfan. Anti-thymocyte globulin (ATG) was used in all recipients of HSCT from unrelated donors. The aplastic anemia patients received cyclophosphamide and ATG.

The Local Ethics Committee approved this study, and all patients and controls gave their informed consent for the study procedures.

### Determination of polymorphisms

Genomic DNA was isolated from whole frozen blood using a QIAamp® Blood mini kit (Hilden, Germany). The SNPs, *CTLA-4*c.49A>G (rs231775) and CT60G>A (rs3087243), were genotyped using the TaqMan®SNP Genotyping Assays C__*2415786_10* and C__*3296043_10*, respectively (Applied Biosystems, Foster City, USA). Genotyping for SNPs was validated using the RFLP technique described by Daroszewski et al. (2009) and by sequencing. The correlation between retyping methods was 99 % for CT60G>A and *CTLA-4*c.49A>G.

### Statistical analyses

The median was used as the location parameter. The *S*_*n*_ statistic was computed as the measure of variability: *Sn* = *med*{*med*|*x*_*i*_ − *x*_*j*_|; *j* = 1 … *n*} (Rousseeuw P.J.and Croux C 1993). A higher *S*_*n*_ value reflects higher variability. Additionally, the first and third quartiles (Q), and minimal and maximal observations were reported. The probability of aGvHD was modeled with a logistic model. The model coefficients and their 95 % confidence intervals (95 % CIs) were estimated based on B = 4900 bootstrap samples. The *R*^2^ coefficient is the fraction of the variation in the response variable explained by the model. The chi-square test, χ^2^_df_, was used to test the null hypothesis that cases and controls have the same distribution of genotype counts. In the case of small numbers, the distribution of the test statistics was estimated numerically. The *odds ratio* (OR) was computed as the measure of effect size. Departure from the Hardy-Weinberg equilibrium (HWE) was tested with the chi-square test. Haplotype frequencies (HFs) among SNPs were estimated with a *maximum likelihood* function (Excoffier and Slatkin 1995). The measure for the estimation of pairwise linkage disequilibrium (LD) was the squared correlation between two SNPs, *R*^2^ (Excoffier and Slatkin 1995). For two SNPs, *r* and *R*^2^ were obtained as $r=\frac{D_{ij}}{\sqrt{p_i{q}_j}}$, where *p*_*i*_ and *q*_*j*_ are the population allele frequencies of the *i*th allele on locus *A* and the *j*th allele on locus *B*, *D*_*ij*_ = *x*_*ij*_ − *p*_*i*_*q*_*j*_ and *x*_*ij*_ is the frequency of the haplotype with alleles *i* and *j* on loci *A* and *B*, respectively, ${R}^2={\displaystyle {\sum}_i^2{\displaystyle {\sum}_j^2\frac{D_{ij}^2}{p,{q}_j}}}$. The likelihood ratio statistic, LRS, was used to test for differences in haplotype frequencies between cases and controls, *LRS* = 2(*LL*_*cases*_ + *LL*_*controls*_ − *LL*_*combined*_).

To control for type I errors in the case of many tests for differences between the SNP genotypes of cases and controls, the adjusted significance level was estimated. Because of the correlation between SNPs, the estimation of α was performed numerically.

## Results

### Acute GvHD

Acute GvHD was diagnosed and graded according to the consensus conference on aGvHD diagnosis (Przepiorka et al. 1995). Five patients died before day 30 after transplantation and were excluded from the analysis. Acute GvHD grades 0, 1, 2, 3, and 4 appeared in 134, 122, 36, 8, and 5 HSCT recipients, respectively. Data were not available for two patients. The detailed characteristics of the recipients’ groups with and without aGvHD are presented in Table 1.

**Table 1:** Clinical characteristics of recipients according to aGvHD presence

Variable		aGvHD (+)		aGvHD (−)		OR	95 % CI
		*N*	%	*N*	%		
Type of HSCT	RD	45	*26.30*	57	*42.50*	0.48	0.29–0.78
	URD	126	73.70	77	57.50		
	Σ	171	100 %	134	100 %	*p* = 0.004	
Conditioning regimen	RIC	9	5.30	20	14.90	2.40	1.65–3.48
	RTMAC	35	20.5	51	38.1		
	MAC	127	74.3	63	47		
	Σ	171	100 %	134	100 %	*p* = 0.00001	
HLA mismatch (MM)	MM =0	125	73.10	115	85.80	2.11	1.22–3.62
	MM =1	41	24.00	18	13.40		
	MM =2	5	2.90	1	0.70		
	Σ	171	100 %	134	100 %	*p* = 0.008	
Donor-recipient sex disparity	F → F	30	17.80	26	19.80	1^a^	–
	F → M	30	17.80	26	19.80	1.00	0.48–2.10
	M → F	51	30.20	34	26.00	1.30	0.66–2.57
	M → M	58	34.30	45	34.40	1.12	0.58–2.15
	Σ	169	100 %	131	100 %	*p* = 0.85	
Relapse	Yes	21	14.00	14	11.40	1.27	0.61–2.61
	No	129	86.00	109	88.60		
	Σ	150	100 %	123	100 %	*p* = 0.52	
Variables		aGvHD (+)		aGvHD (−)		Medians difference^d^	95 % CI
Recipient age	Q1^b^	26.50		27.00		−3.92	−8.07–0.14
	Median	33.00		37.00			
	*S*_*n*_^c^	10.00		11.00			
	Q3^b^	45.00		48.00		*p* = 0.11	
Donor age	Q1	26.00		24.00		1.87	−1.84–5.73
	Median	33.00		32.00			
	*S*_*n*_	10.00		10.00			
	Q3	42.00		41.80		*p* = 0.36	
Overall survival for died recipients	Q1	2.90		2.52		−1.59	−4.64–2.65
	Median	5.43		6.92			
	*S*_*n*_	3.95		6.58			
	Q3	12.64		14.90		*p* = 0.35	
Overall survival for living recipients	Q1	22.16		23.36		3.73	−6.26–12.89
	Median	35.80		31.97			
	*S*_*n*_	17.36		14.60			
	Q3	53.00		43.76		*p* = 0.42	

*aGvHD* acute graft versus host disease, *OR* odds ratio, *CI* confidence interval, *HSCT* hematopoietic stem cell transplantation, *HLA* human leukocyte antigen, *RD* related donor, *URD* unrelated donor, *RIC* reduced-intensity conditioning, *RTMAC* reduced toxicity myeloablative conditioning

^a^Baseline group

^b^Q1 and Q3: first and third quartiles

^c^ *S*_*n*_ is the measure of variability

^d^Case-control bias-corrected difference

In a univariate analysis, we examined the potential prognostic variables, including the type of transplantation (RD or URD), HLA matching, recipient age, donor age, sex mismatch, conditioning regimen, and diagnosis. We found strong associations between three variables and the risk of aGvHD: the HLA matching, the conditioning regimen, and the type of allogeneic HSCT. As expected, a disparity in HLA matching was strongly associated with an increased risk of aGvHD (OR 2.11; 95 % CI 1.22–3.62; *p* = 0.008). In addition, the conditioning regimen used for HSCT was associated with the aGvHD risk. When we assumed RIC conditioning as less associated with aGvHD risk, then the OR for RTMAC was 1.50, whereas for MAC, the OR was 4.50. In total, the aggressiveness of the conditioning regimen was associated with the aGvHD risk with an OR of 2.40 (95 % CI 1.65–3.48; *p* = 0.00001). The RD HSCT was associated with approximately a twofold decrease in risk of aGvHD (OR 0.48; 95 % CI 0.29–0.78; *p* = 0.004) compared to URD HSCT.

The sex of donors and recipients did not influence aGvHD (*p* = 0.85). The OR was similar for four donor-recipient sex combinations (F → F, F → M, M → F, M → M). The age of the donor and recipient had no impact on the aGvHD risk. The average overall survival was similar in groups of patients with and without aGvHD.

#### A CTLA-4 gene polymorphism study

No polymorphism data from donors and recipients demonstrated any deviation from the Hardy-Weinberg equilibrium. We have observed weak linkage disequilibrium between the *CTLA-4*c.49A>G and CT60G>A polymorphisms in donors (*R*^2^ = 0.49) and in recipients (*R*^2^ = 0.524). Haplotype analysis showed that only three haplotypes were observed among the donors and recipients. The frequencies were G-G (45.80 and 41.30 %), A-A (35.60 and 37.20 %), and A-G (18.60 and 21.50 %).

#### An analysis of the associations between the CTLA-4 gene polymorphisms and aGvHD

In a univariate analysis, we found that none of donor polymorphisms were associated with susceptibility to aGvHD (Table 2), although we observed a lower frequency of disease in those recipients who were transplanted from the CT60G>A[AA] donors than in recipients transplanted from donors possessing the CT60G>A[G+] allele (9.50 vs. 17.30 %).

**Table 2:** Genotypes frequency of donors’ *CTLA-4* SNPs in relation to aGvHD presence

Donors polymorphisms		aGvHD (+)		aGvHD (−)		OR	95 % CI	
		*N*	%	*N*	%			
*CTLA-4*c.49A>G (rs231775)	AA	54	32.10	47	35.10	1^a^	–	χ^2^_df=2_ = 0.49 *p* = 0.78
	AG	87	51.80	64	47.80	1.18	0.71–1.96	
	GG	27	16.10	23	17.20	1.02	0.52–2.02	
	Σ	168	100 %	134	100 %	–	–	
	HWE	*p* = 0.43		*p* = 0.86				
CT60G>A (rs3087243)	GG	69	41.10	47	35.30	1^a^	–	χ^2^_df=2_ = 4.16 *p* = 0.13
	GA	83	49.40	63	47.40	0.90	0.55–1.47	
	AA	16	9.50	23	17.30	0.48	0.23–1.00	
	Σ	168	100 %	133	100 %	–	–	
	HWE	*p* = 0.23		*p* = 0.86				

*aGvHD* acute graft versus host disease, *OR* odds ratio, *CI* confidence interval, *HWE* Hardy-Weinberg equilibrium

^a^Baseline group

For recipients with the CT60G>A polymorphism, we found that it was significantly associated with aGvHD risk (Table 3). The distribution of genotypes of recipients with the CT60G>A polymorphism was different in recipients with and without aGvHD (χ^2^_df=2_ = 9.27, *p* = 0.01, *p*_corected_ = 0.04), and the presence of at least one A allele decreased the risk of aGvHD about twofold [for CT60G>A[GA], the OR was 0.47 (95 % CI 0.28–0.79), and for CT60G>A[AA], the OR was 0.45 (95 % CI 0.21–0.98)].

**Table 3:** Genotype frequency of recipients’ *CTLA-4* SNPs according to aGvHD presence

Recipients polymorphisms		aGvHD (+)		aGvHD (−)		OR	95 % CI	
		*N*	%	*N*	%			
*CTLA-4*c.49A>G (rs231775)	AA	33	23.60	38	30.60	1^a^	–	χ^2^_df=2_ = 3.82 *p* = 0.15
	AG	76	54.30	69	55.60	1.27	0.72–2.23	
	GG	31	22.10	17	13.70	2.07	0.98–4.36	
	Σ	140	100 %	124	100 %	–		
	HWE	*p* = 0.40		*p* = 0.14				
CT60G>A (rs3087243)	GG	69	49.60	39	31.20	1^a^	–	χ^2^_df=2_ = 9.27 *p* = 0.04^b^
	GA	55	39.60	67	53.60	0.47	0.28–0.79	
	AA	15	10.80	19	15.20	0.45	0.21–0.98	
	Σ	139	100 %	125	100 %	–	–	
	HWE	*p* = 0.43		*p* = 0.36				

*aGvHD* acute graft versus host disease, *OR* odds ratio, *CI* confidence interval, *HWE* Hardy-Weinberg equilibrium

^a^Baseline group

^b^ *p* value adjusted to number of tests

Logistic regression analysis was used to test whether the association observed between the recipient CT60G>A[GG] genotype and susceptibility to aGvHD was influenced by other factors, including HLA matching, conditioning regimen (MAC, RTMAC, or RIC), graft source (BM or PBPC), mode of transplantation (related or unrelated donor), diagnosis, recipient age, donor sex, and age. Both recipient and donor genotypes were included in the analysis to exclude any possible interaction between them. The model, including conditioning regimen, type of transplantation (RD vs. URD), and the recipient CT60G>A polymorphism, fitted the best to the probability of aGvHD disease.

In this model, the OR increased 2.68-fold from RIC to RTMAC and from RTMAC to MAC therapy (95 % CI 1.65–4.07, *p* = 0.00003). Transplantation from an unrelated donor raised the risk of aGvHD 1.87-fold (95 % CI 1.02–3.24, *p* = 0.04) compared to RD HSCT.

Moreover, the recipient CT60G>A[GG] genotype was associated with a 2.63 higher aGvHD risk (95 % CI 1.45–4.59, *p* = 0.001) than that of CT60G>A[GA] and CT60G>A[AA]. This means that a recipient possessing at least one A allele had approximately a twofold reduced chance of aGvHD compared with a recipient with CT60G>A[GG] who underwent the same type of transplantation and conditioning regimen.

The expected frequency of aGvHD in relation to the conditioning regimen, the type of HSCT, and the recipients’ CT60G>A genotype are presented in Table 4. The lowest aGvHD risk group consisted of recipients possessing the CT60G>A[A+] allele who received a transplant from related donors (RD HSCT) with RIC conditioning (CT60G>A[A+]/RD-HSCT/RIC), and it was chosen as the baseline (reference group) for OR calculations.

**Table 4:** The best regression model of probability of aGvHD syndrome including the type of HSCT donor (related (RD) vs. unrelated (URD)), the conditioning regimen (reduced-intensity conditioning (RIC), reduced-toxicity myeloablative conditioning (RTMAC), and mieloablative conditioning (MAC)), and recipient genotype

Variable^a^	OR	95 % CI	*p* value
Conditioning regimen RIC → RTMAC → MAC	2.68	1.65–4.07	0.00003
URD HSCT	1.87	1.02–3.24	0.04
Recipient’s genotype CT60G>A[GG]	2.63	1.45–4.59	0.001
χ^2^_df=3_ = 38.4, *p* < 0.00001 *R* ^2^ = 0.15			

β_0_ = −3.19

*OR* odds ratio, *CI* confidence interval, *HSCT* hematopoietic stem cell transplantation

^a^CT60G>A[A+] + RD HSCT+ RIC as baseline

The estimated aGvHD frequency in the reference group of recipients was 10.50 %. The expected frequency of aGvHD for recipients with the predisposing CT60G>A[GG] genotype transplanted from a related donor and with the RIC conditioning regimen was 23.40 % with an OR of 2.60.

The estimated aGvHD frequency in the recipients with CT60G>A[A+] transplanted from related donors and treated with RTMAC gained 23.40 % compared with the reference group with an OR of 2.60 (conditioning regiment effect). In the CT60G>A[GG] recipients with RD HSCT and RTMAC preparation, the frequency increased to 44.3 %, with the raised OR being 6.78 (additive effect of the recipients’ genotype and the use of RTMAC), whereas in the recipients with the CT60G>A[GG] genotype transplanted from unrelated donors with the use of RTMAC, the aGvHD expected frequency was 59.40 % and the OR increased to 12.47 compared with the reference group.

Consequently, in the highest risk group of recipients with the predisposing CT60G>A[GG] genotype, transplants from unrelated donors, and the MAC regimen, the expected frequency of aGvHD was 79.2 % and the OR was as high as 32.46 compared with the reference group (CT60G>A[A+]/RD-HSCT/RIC) (Table 5).

**Table 5:** Development of aGvHD syndrome in group of HSCT recipients in relation to (A) the type of HSCT donor (related (RD) vs. unrelated (URD)), the conditioning regimen (reduced-intensity conditioning (RIC), reduced-toxicity myeloablative conditioning (RTMAC), and mieloablative conditioning (MAC)) and the recipient’s genotype (CT60G>A[GG]); (B) the type of HSCT donor (related vs. unrelated), and the recipient’s genotype (CT60G>A[GG]); and (C) the recipient’s genotype (CT60G>A[GG])

A	Type of HSCT	R CT60G>A[GG]	RIC			
			Yes	No	% Yes	OR^a^
	RD	GG	1	7	23.40	2.60
		GA/AA	3	3	10.50	1.00
	URD	GG	3	2	35.90	4.77
		GA/AA	0	8	17.80	1.85
	Total		7	20	20.00	1^b^
	Type of HSCT	R CT60G>A[GG]	RTMAC			
			Yes	No	% Yes	OR^a^
	RD	GG	2	6	44.30	6.78
		GA/AA	3	13	23.40	2.60
	URD	GG	15	10	59.40	12.47
		GA/AA	10	19	36.00	4.79
	Total		30	48	41.10	2.79^b^
	Type of HSCT	R CT60G>A[GG]	MAC			
			Yes	No	% Yes	OR^a^
	RD	GG	14	6	67.40	17.62
		GA/AA	14	17	44.30	6.78
	URD	GG	33	8	79.20	32.46
		GA/AA	38	26	59.40	12.47
	Total		99	57	63.50	6.96^b^
B	Type of HSCT	R CT60G>A[GG]	Yes	No	% Yes	OR^c^
	RD	GG	17	19	52.20	2.28
		GA/AA	20	33	32.40	1.00
	URD	GG	51	20	70.40	4.96
		GA/AA	48	53	47.70	1.90
C	R CT60G>A[GG]		Yes	No	% Yes	OR^d^
	GG		68	39	65.90	2.63
	GA/AA		68	86	42.30	

Percent of cases (% Yes) and odds ratios (ORs) are estimated based on model

*HSCT* hematopoietic stem cell transplantation

^a^RD & GA/AA & RIC as baseline

^b^Total RIC as baseline

^c^RD & GA/AA as baseline

^d^GA/AA as baseline

The effect of the recipient CT60G>A genotype was independent of the donor *CTLA-4* polymorphism and the age of the recipients and donors (*p* = 0.39).

The haplotype distribution between groups of recipients with and without aGvHD was different (χ^2^_df=2_ = 9.60; *p* = 0.008). Haplotypes G-G and A-G were associated with an increased risk of aGvHD, whereas haplotype A-A was protective against aGvHD (Table 6).

**Table 6:** Haplotypes frequency of *CTLA-4* gene polymorphisms among HSCT recipients in relation to aGvHD

*CTLA-4*c.49A>G	CT60G>A	aGvHD (+)	aGvHD (−)	RR^a^
G	G	50.00	41.80	1.20
A	A	29.30	42.20	0.69
A	G	20.70	16.00	1.29
χ^2^_df=2_ = 9.60; *p* = 0.008		100 %	100 %	–

*aGvHD* acute graft versus host disease, *HSCT* hematopoietic stem cell transplantation

^a^RR—relative risk cases/controls

#### An analysis of the associations between the CTLA-4 gene polymorphisms and relapse

Relapse occurred in 34 patients, and 27 of them died due to this complication. None of donors’ and recipients’ genetic variations and clinical parameters affected the incidence of relapse (data not shown).

#### An analysis of the associations between the CTLA-4 gene polymorphisms and overall survival

The median follow-up time was 21.27 months (range, 0.5–67.5 months). In the studied cohort of patients, 201 recipients lived and 111 died during the observation period. The median overall survival (OS) for all patients was 23.67 months (quadrille (Q), Q1 = 8.45; Q3 = 40.20). For living patients, the median OS was 34.23 months (Q1 = 23.36; Q3 = 50.38), whereas for dead recipients, the median OS was 5.53 months (Q1 = 2.77; Q3 = 13.48).

None of the donors’ and recipients’ genetic factors and clinical parameters had an impact on the overall survival (*p* = 0.49).

## Discussion

In early immunogenetic studies in transplantation, genes encoding HLA were the first targets for donor-recipient matching because the HLA molecules are crucial for T cell recognition. During the last several years, growing attention has been focused on polymorphisms in genes of the KIR family (Gagne et al. 2002;Giebel et al. 2009;Ludajic et al. 2009), as well as on cytokine and cytokine receptor genes (Bogunia-Kubik 2004;Dickinson and Holler 2008;Karabon et al. 2005;Markey et al. 2008).

Given the importance of co-stimulatory pathways and attempts to use CTLA-4-Ig to prevent GvHD in animal models (Blazar et al. 1996;Ichiki et al. 2006), several published reports evaluated the association between donor *CTLA-4* gene polymorphisms and outcome after hematopoietic stem cell transplantation (Bosch-Vizcaya et al. 2012;Iravani-Saadi et al. 2014;Mossallam and Samra 2013;Perez-Garcia et al. 2009;Piccioli et al. 2010). However, the results presented in these studies were inconsistent. Predominantly two polymorphisms in the *CTLA-4* gene were investigated, *CTLA-4*c.49A>G and CT60G>A.

The first and the largest study performed on a Spanish population by Perez Garcia et al. (Perez-Garcia et al. 2007) indicated that the *CTLA-4*c.49A>G SNP in RD-HSCT donors had no impact on aGvHD development, whereas the donor CT60G>A[AA] genotype was associated with a higher risk of aGvHD II-IV but with a better 5-year OS and a lower risk of relapse. The Azarian et al. study (Azarian et al. 2007) showed no association between the donor *CTLA-4*c.49A>G and CT60G>A SNPs and susceptibility to aGvHD in RD-HSCT in a French population; however, the authors found that the presence of the G allele in those SNPs increased the risk of developing chronic GvHD. Based on a study of a Tunisian population, Sellami et al. (Sellami et al. 2011) postulated that the haplotype *CTLA-4* g.319C>T[C]/*CTLA-4*c.49A>G[G] was associated with the incidence of chronic GvHD. The Vannucchi et al. study (Vannucchi et al. 2007) devoted to URD HSCT showed no association between *CTLA-4*c.49A>G and acute or chronic GvHD, but like the Perez–Garcia et al. study (Perez-Garcia et al. 2007), it indicated that the CT60G>A[AA] genotype raised the risk of severe aGvHD and cGvHD. Similarly, Chien et al. (Chien et al. 2012) pointed out an association between the CT60G>A polymorphism and severe aGvHD risk in unrelated bone marrow transplantation. Different results were obtained by Xiao et al. for a Chinese population (Xiao et al. 2012). They found that patients receiving stem cells from related donors with the CT60G>A[AA] polymorphism had a lower risk of aGvHD grades II–IV.

In contrast, other studies performed on RD HSTC or URD HSCT in different populations (Spanish, Japanese, and Italian) reported no association between those polymorphisms and the aGvHD risk but indicated an effect of those SNPs on the overall survival or relapse rate (Bosch-Vizcaya et al. 2012;Jagasia et al. 2012;Piccioli et al. 2010).

In our study, we found no impact of donor *CTLA-4*c.49A>G and CT60G>A polymorphisms on HSCT outcome. Like Xiao et al. (Xiao et al. 2012), we observed a not statistically significant lower frequency of the donor CT60G>A[AA] genotype than that of the CT60G>A[GG] genotype in patients with aGvHD.

The reasons for these inconsistencies may include a different type of transplantation, a different conditioning regimen, GvHD prophylaxis, and ethnic background. For example, the frequency of the CT60G>A[GG] genotype in Polish donors is approximately 40.00 %, but it is approximately 25.00 % in Spanish donors (Perez-Garcia et al. 2007). Moreover, in the Polish population, the frequency of the [GG] genotype is higher than that of the [AA] genotype, whereas in the Spanish population a different relationship was observed. Furthermore, in our analysis, we compared the group without aGvHD symptoms (aGvHD grade 0) to the group with any aGvHD symptoms (grades I–IV).

CTLA-4 expressed on donors’ T cells plays the crucial role in the regulation of the immune response, in particular, in the graft versus leukemia effect and in controlling infections; therefore, the majority of studies have focused on the donor *CTLA-4* gene polymorphism. However, a limited number of studies have highlighted the role of the *CTLA-4*c.49A>G and CT60G>A polymorphisms in recipients. Orru et al. (Orru et al. 2012) showed that in URD transplantation for thalassemia patients from Italy, the recipient CT60G>A[AA] genotype was associated with a higher risk of aGvHD grade II or higher. In addition, Picciolli et al. (Piccioli et al. 2010) found that the recipient *CTLA-4*c.49A>G but not CT60G>A was a risk factor for aGvHD in an Italian population, whereas Xiao et al. (Xiao et al. 2012) in a Chinese population and Mosssallam et al. in a Tunesian population (Mossallam and Samra 2013) found no effect of the recipient *CTLA-4*c.49A>G and CT60G>A SNPs on aGvHD risk.

In the present study, we showed by a univariate analysis that the CT60G>A[GG] genotype was associated with approximately a twofold higher risk of aGvHD compared with the CT60G>A[GA] and CT60G>A[AA] genotypes. The multivariate analysis, which included known aGvHD risk factors, showed that the CT60G>A[GG] genotype, together with the type of transplantation and aggressiveness of the conditioning regimen, was an independent risk factor for this complication. We found that patients who were recipients of the CT60G>A[GG] genotype transplanted from unrelated donors and who received the myeloablative conditioning regimen had more than a 30-fold higher risk of aGvHD than patients who received at least one A allele transplanted from related donors and who received a reduced toxicity conditioning regimen.

The presence of the G allele of both the *CTLA-4*c.49A>G and CT60G>A polymorphisms has been reported to be associated with the risk of many autoimmune diseases, including coeliac disease, systemic lupus erythematosus, multiple sclerosis, chronic inflammatory arthropathies, Sjogren syndrome, and autoimmune thyroiditis (Gough et al. 2005;Kavvoura et al. 2007;Kavvoura and Ioannidis 2005).

Here, we postulate that the presence of the CT60G>A[GG] genotype in recipients is associated with an impaired regulation of T cell activation and that it promotes the development of aGvHD.

On the basis of current knowledge, it is difficult to explain the role of the recipient *CTLA-4* gene polymorphisms in the development of aGvHD. One possible explanation is that the CTLA-4 molecules present on surviving host T cell populations, including recipient lymphocytes, leukemic monocytes, and especially T reg cells, may contribute to the clinical outcome after HSCT (Bayer et al. 2009;Laurent et al. 2010;Pistillo et al. 2003). Moreover, our functional study showed that the recipients’ pretransplant CTLA-4 mRNA level and protein expression were associated with aGvHD risk (Karabon et al. 2015).

The limitation of this study is the heterogenic group of donor-recipient pairs; however, we believe that the implementation of a multivariate logistic regression analysis, which includes other prognostic factors for aGvHD development, allows us to overcome this weakness of the study.

Probably due to the low relapse rate observed in our cohort of patients, we were not able to find any association between donor and recipient *CTLA-4* gene polymorphisms and aGvHD. We also did not find any association between the SNPs investigated here and OS. The data presented in the literature regarding the association of *CTLA-4*c.49A>G and CT60G>A with relapse risk and overall survival were inconsistent, which was clearly summarized by Mossallam et al. (Mossallam and Samra 2013).

## Conclusion

Our data suggest that the recipient CT60G>A[GG] genotype has an impact on aGvHD development, especially in patients who receive transplants from unrelated donors and who receive myeloablative conditioning.
